# Assessment of Coverage and Quality of Selected Clinical Chemistry Tests among Medical Laboratories of Health Facilities in Jimma Zone, South West Ethiopia

**DOI:** 10.1155/2019/5954313

**Published:** 2019-03-03

**Authors:** Aklilu Getachew, Waqtola Cheneke, Yaregal Asres, Shiferaw Bekele, Estifanos Kebede

**Affiliations:** School of Medical Laboratory Science, Faculty of Health Sciences, Institute of Health, Jimma University, Jimma, Ethiopia

## Abstract

**Background:**

Medical laboratories play essential roles in measurement of analyte in clinical sample for the diagnosis and monitoring of diseases. Thus, data generated from the laboratory have to be reliable for which strict quality assurance is maintained.

**Objective:**

To assess the coverage and quality of selected clinical chemistry tests among medical laboratories of health facilities in, Jimma Zone, South West Ethiopia.

**Methods:**

A cross-sectional study was conducted at Jimma Zone on health facilities from August 15 to September 15, 2014. Eighty-six health facility laboratories were included in the study. We classified laboratories into laboratories with clinical chemistry service and those without clinical chemistry service clusters and those with clinical chemistry laboratory were again clustered according to their level. Data were collected by direct observation, interview, and proficiency testing (PT). The collected data were analyzed and compared with CLIA PT goal for TEa by considering total allowable error ± 20%, ±10%, ±15%, and ±20 for each analyte, ALT, glucose, creatinine, and total bilirubin, respectively.

**Result:**

From total of 86 health facilities with laboratories, 23.3% (n=20) had clinical chemistry service, of which 77.2% results were reported outside of the allowable error limit.

**Conclusion:**

According to this study the availability of clinical chemistry test service was very minimal and facilities giving the service do not fulfill the minimum standard for quality; thus clients were either getting wrong clinical decision or misdiagnosed. Therefore, the external and internal quality assessment programs should be reviewed very well. Those laboratories whose report was outside of the allowable error should have to report results with the appropriate reference range so that physicians consider that. Establishment of local clinical chemistry reference range can also minimize the problem.

## 1. Introduction

Clinical laboratory services are an essential component in the health care system, which aids the diagnosis and treatment of infectious and noninfectious diseases [1]. One of the areas in this regard is clinical chemistry laboratory service. Although the laboratory service has its importance in the health care system, poor quality of laboratory results can lead to inappropriate clinical decision. The importance of quality laboratory result in the health care system is well recognized globally. However quality laboratory result in all clinical laboratories, including clinical chemistry, remains in their nascent stages in most African countries. Being cognizant of these weaknesses, the World Health Organization Regional Office for Africa WHO/AFRO strategic direction priorities for 2010–2015 highlighted the importance of laboratory quality services through partnerships and harmonization of technical support to accelerate actions on human immune virus/acquired immune deficiency syndrome (HIV/AIDS), malaria, and tuberculosis [2].

In Ethiopia it is Ethiopia Public Health Institute (EPHI) which is working with regions, partners, and stakeholders to coordinate efforts on bringing each tier of the network up to the standards. In order to do so, EPHI conducts the annual assessment of health facility laboratories, in coordination with the quality program, which were utilized in the context of identifying gaps between the state of laboratories and the national standards [3].

The current laboratory system in Ethiopia is the same with the health care system in the country, while clinical chemistry test service is available only in health centers and higher leveled health facilities [4]. Diagnostic laboratories including clinical chemistry service are the main components of these facilities from the health center level upwards, although the size and complexity of the laboratory differ at each type of facility [5].

Even though this system is in place, most of the services mentioned above are not being undertaken even at hospital level. Moreover, quality is highly under question mark in those facilities which are giving the service; quality laboratory service in Ethiopia is still a challenge [6]. This is because quality laboratory service is not only designing structures for quality but it should also guarantee that each and every step in the total testing process (TTP) is correctly performed through implementation of quality assurance program [7]

The government of Ethiopia has done some for the quality improvement in laboratories and it is also usual to hear from different advertising agencies on quality laboratory service, but still it is our day to day experience to come across patients and physician's rumor about the quality service they are receiving from laboratories [8]. In addition to the existence of laboratory quality related problems mentioned above, the magnitude is unknown that the coverage and performance of medical laboratories of health facilities in Jimma Zone have not been reported so far. Therefore, the aim of this study was to assess the coverage and quality of selected clinical chemistry tests among medical laboratories of health facilities in, Jimma Zone, South West Ethiopia.

## 2. Methods and Materials

### 2.1. Study Area and Seating

The study was conducted in Jimma Zone, Oromia Regional State, and South West Ethiopia from August 15 to September 15, 2014. A total of 766 health facilities were found in Jimma Zone; from those 36 were in Jimma City Administration. From the total health facilities 4 were governmental hospitals, 110 health centers, 486 health posts, 28 higher private clinics, 128 medium private clinics, 2 other governmental hospitals, 4 nongovernmental clinics, and 4 diagnostic laboratories. A cross-sectional study design was conducted in all health facilities that had been providing laboratory service in Jimma Zone.

From the total health facilities 86 were giving laboratory service. For the purpose of this study, health facilities with laboratory service were classified into with and without clinical chemistry test service. Again, those health facilities providing clinical chemistry test service were grouped into five depending on their level, namely, government hospitals, government health centers, private clinics, none governmental organization (NGO) health facilities, and diagnostic laboratories.

### 2.2. Data Collection Method and Instrumentation

#### 2.2.1. Questionnaire Based Interview

We used questionnaire based interview in all health facilities with laboratory service in the zone to check for the availability of clinical chemistry test service and the reason for nonavailability. The type of analyzer and the educational level of the laboratory professional were asked, if they were giving clinical chemistry test service.

#### 2.2.2. Onsite Supervision

We used Stepwise Laboratory Improvement Process towards Accreditation checklist for visually inspecting the laboratories. The checklist contains a total of 111 main sections, 334 questions, and 258 points [7]. Each item has been awarded a point value of 2, 3, 4, or 5 points based on the relative importance and/or complexity. Rating to all questions was being yes, partial, or no. The distribution of the observation score and result obtained was compared to predetermined statistical parameters that show whether the observations are under the expected level of standard.

#### 2.2.3. Proficiency Testing

The laboratories invited in proficiency testing scheme include 2 government hospitals, 2 higher private clinics, 3 medium private clinics, 2 diagnostic laboratories, and 1 other governmental health institution. Every laboratory was expected to do 3 alanine aminotransferase (ALT), 3 glucose, 3 creatinine, and 3 total bilirubin from both normal and pathological control samples.

Those four clinical chemistry test parameters (ALT, glucose, creatinine, and total bilirubin) were selected so that we can cover the four principles of clinical chemistry. First we predetermined control samples for proficiency testing. Twelve vials of commercially available lyophilized control samples were used for both normal and pathological control samples. The control samples were prepared by reconstituting it with distilled water. Each vial of the control sample was taken from the refrigerator, allowed to stay at room temperature, and reconstituted and mixed very well. From each vial of the control sample, we made 20 times test run on both normal and pathological control PT samples for the four clinical chemistry test panel (ALT, glucose, creatinine, and total bilirubin) so that we can determine the level of stated value allowable error under optimized condition and we took the ± 20%, ±10%, ±15%, and ±20 for each analyte, ALT, glucose, creatinine, and total bilirubin, respectively.

Then, after the standardization we took 0.6ml control sample, aliquoted into Nunc tube, and each Nunc tube was labeled randomly. The 0.6 ml of control samples was calculated by considering the total amount of control samples required for the four clinical chemistry tests parameters to be assessed in each clinical chemistry test performing health facilities.

Ten participating health facilities performing clinical chemistry tests were allowed to perform three tests for the four selected clinical chemistry test parameters for both pathological and normal control samples.

Thus each health facility performed 24 tests; a total of 240 clinical chemistry tests were performed. For all of the participating laboratories the result of the control samples provided were unknown by investigators. The quality checking was by considering those health facilities with test result out of ± 20%, ±10%, ±15%, and ±20 for each analyte, ALT, glucose, creatinine, and total bilirubin, respectively; allowable limit of error was taken as inaccurate and imprecise whereas those within the allowable error were taken as accurate and precise.

### 2.3. Data Quality Management

The control samples dispatched to participating laboratories were prepared strictly following the manufacturer's instruction. The reconstituted samples were randomly labeled. For ten proficiency testing (PT) participating laboratories, control samples were given blindly by randomly selecting. In order to maintain their stability, the prepared samples were stored at -20 degree centigrade before being transported to participating laboratories. We used cold chain system when PT samples were transported to the participating laboratories.

## 3. Results

A total of 86 health facilities were included in this study, from which 50 (58%) and 36 (42%) were found in the rural and urban part of the zone, respectively. Out of the total participated health facilities only 20 (23%) were giving clinical chemistry service; all were found in Jimma City; no laboratory outside of Jimma city in the zone was providing clinical chemistry test service.

Out of 66 health facilities without clinical chemistry tests, it was found that 50 (75%) were governmental. The main reason for these laboratories not to undertake the clinical chemistry test service was lack of money which accounts 63 (95%) (Table 1)

According to the quality assessment score performed on those health facilities all of them scored below 50 from the expected maximum score of 258. This showed that no laboratory has scored the least score 1 star (143-165pts). The maximum score by the laboratories was 40-49 points by 4(20%) laboratories and the least was between 10 and 19 points which were scored by 7 (35%) laboratories.

This study has also showed that most 8 (40%) of the facilities utilize linear company chemistry analyzer, while 1 health facility utilizes Dr. Lange laboratory system manual chemistry analyzer (LP450) and pentra 400 chemistry analyzer. Most of the participating health facilities utilize linear reagents 10 (50%) while ABX pentra reagent was used by 1 health facility.

Regarding the educational status of the employees the highest level of education was MSc holder which was found in only one of the health facilities. Most of clinical chemistry service giving health facilities 16 (80%) were owned by private (Table 2)

From the expected 240 test results on the scheme 216 test results were reported. Few tests of total bilirubin and creatinine, 23(95.8%), were not tested by some of the participating laboratories which, followed by ALT which accounts 1 (4.2%).

Normal range, stated value, mean of participating laboratories, acceptable values based on ± 20%, ±10%, ±15%, and ±20 for each analyte, ALT, glucose, creatinine, and total bilirubin, respectively, of the stated value, and manufacturer provided range values for each pathological and normal control sample were summarized on Tables 3 and 4.

**Figure 1:**
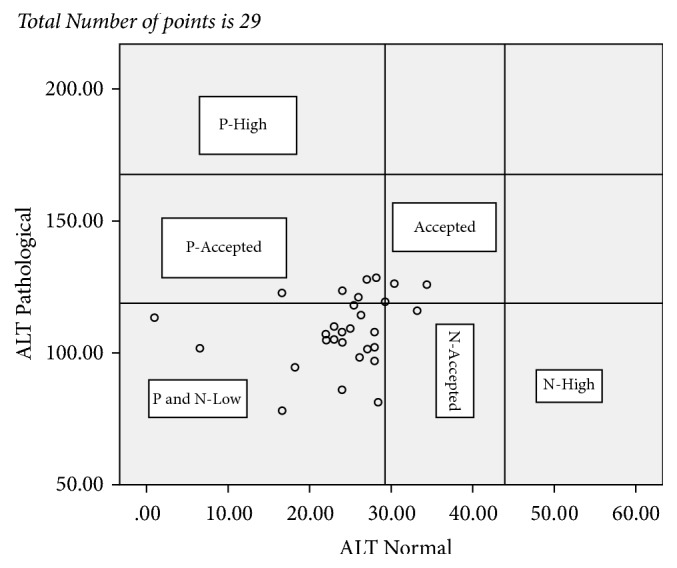
Plot of control sample ALT pathological P values versus control sample ALT normal N values for the total of 29 reported points. P: pathological; N: normal.

**Figure 2:**
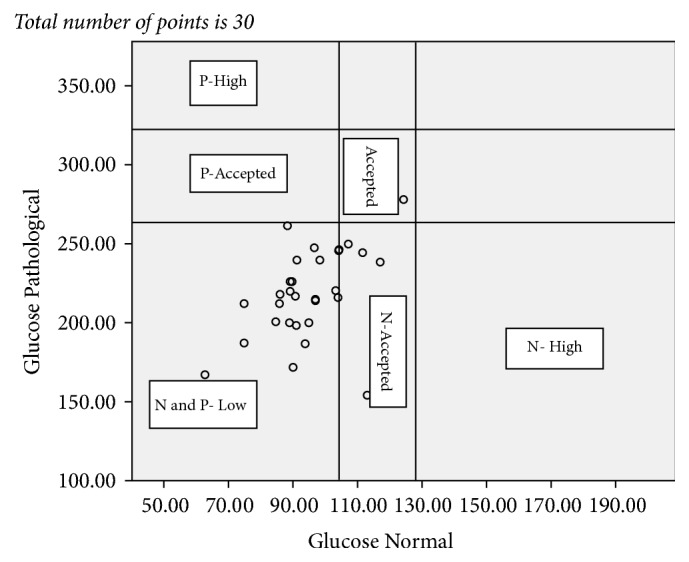
Plot of control sample glucose pathological P values versus control sample glucose normal N values for the total of 29 reported points. P: pathological; N: normal.

**Figure 3:**
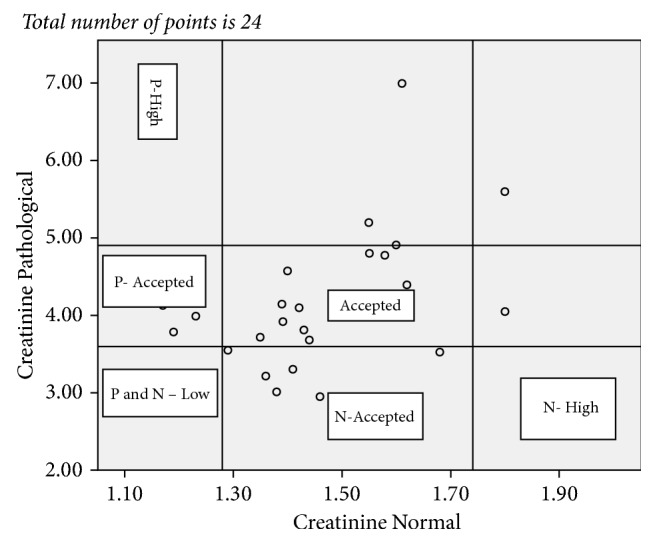
Plot of control sample creatinine pathological P values versus control sample creatinine normal N values for the total of 29 reported points. P: pathological; N: normal.

**Figure 4:**
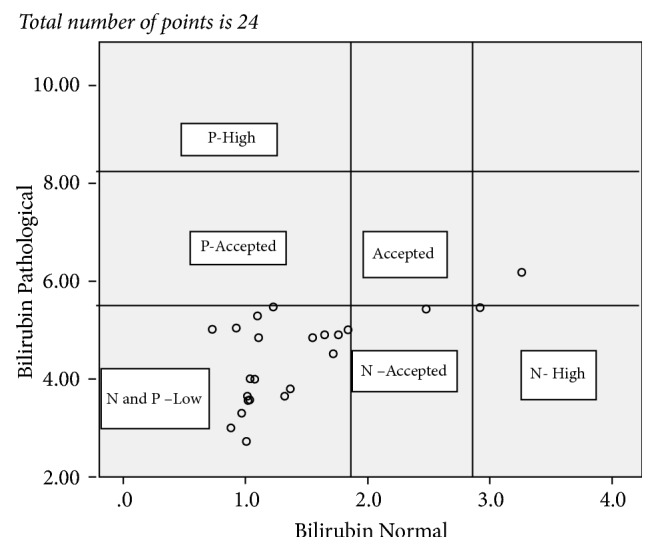
Plot of control sample total bilirubin pathological P values versus control sample total bilirubin normal N values for the total of 23 reported points. P: pathological; N: normal.

The result of all the test values reported from the 10 laboratories and statistical relations were summarized in Table 5 and Figures 1–4. Figures 1–4 showed not only the actual values obtained but also the correlation between the sets of values. The vertical and horizontal lines are drawn to outline the acceptable ranges (based on the stated values).

Each point on the scatter diagram represents laboratory results provided by PT participating facilities.

From the reported values for pathological ALT 70% of the reported values fall below the acceptable range based on the stated value and no values fall above acceptable range. This means that there is 70% total error falling out of the acceptable range of values. For normal ALT 11.0% of the test result fall below the acceptable range and no test result fall above the acceptable range of the stated value, which makes a total of 11.0% of the results fall out of the acceptable range of the stated value (Figure 1).

From the reported value for the pathological glucose test 96.7% of them fall below the acceptable range according to the stated values and no result falls above the acceptable making a total error of 96.7%. For the normal glucose test 83.3% of the reported value falls below the acceptable range according to the stated values and no result falls above the acceptable range which makes a total error of 83.3% (Figure 2).

For pathological creatinine test 25.0% of the reported value falls below and 12.5% above the acceptable range according to the stated value which makes a total error of 37.5%. For the normal creatinine test 12.0% of the reported value falls below and 8.0% of the reported value falls above the acceptable range according to the stated value which makes a total error of 20.0% (Figure 3).

Similarly for the pathological total bilirubin 96% of the reported value falls below and none of the reported value falls above the acceptable range which makes a total error of 96.0% of the reported value fall out of the acceptable range. For the normal total bilirubin test 88.0% of the reported value falls below and 8.3% fells above the acceptable range which makes a total error of 96.3% of the reported value falling out of the acceptable range (Figure 4).

## 4. Discussion

The main purpose of this study was to show the coverage and quality of clinical chemistry tests in Jimma Zone. During the study it was found that in almost all of the health facility in rural part of the zone there was no health facility which was giving clinical chemistry laboratory service even at the higher (hospital) level. Out of the participated 50 laboratories from rural part of the zone none of them undertake the clinical chemistry test, but according to the national guideline from EPHI [8] even those health facilities at the level I (health centers) should have to conduct AST, creatinine, and glucose test service for the community. However none of the health centers in the rural part of the zone were giving the service. In urban part of the zone from 36 health facilities with laboratory service, it was found that 20 give clinical chemistry test service.

This study has also indicated that the main reason for the laboratories not to undertake clinical chemistry test service was lack of financial efficiency to employ personnel and to purchase chemistry machines and reagents.

However in the city most of the health facilities have laboratory with clinical chemistry test service which means around 55.5% of the health facility from the city undertake clinical chemistry tests; however all of the facilities are giving below standard service. Moreover none of the health facilities with clinical chemistry test service has scored the minimum score of 142 points from the expected score of 258 points. Only 4 (10%) laboratories have scored 40-49, which was also below star 1 according to the SLIPTA guideline provided by WHO [1].

Similarly from 20 health facilities which are giving clinical chemistry test service 10 laboratories have participated in the proficiency testing scheme and all of the participating laboratories in this assessment scheme completed the survey except some laboratories which participated in the proficiency testing scheme do not complete the hole of the test on the test menu because of lack of reagent and sudden failure of instrument. During the study 167 results (77.2%) from both pathological and normal control sample were reported out of the allowable error from the reported 216 tests results in the scheme.

It was also found that there was a lack of accuracy and precision in laboratories test result, which was much greater as compared to the finding in the study done on the quality of liver and kidney function tests in Amhara Regional State, where 65% of the test results fall outside of the allowable range [6].

The medical laboratory surveys in the US classified a laboratory as performing satisfactorily only when 10% or less of the reported values fall out of the allowable limits of error (80% of the result should be within the limit of allowable error) [9]. However, the majority of medical laboratories in Jimma Zone which were giving the service were found to be out of the standards even according to the old Good Clinical Laboratory Practice [10]. Some of the laboratories also did not have adequate reagents and chemicals to do all the necessary tests and thus health decisions could be possibly made based on incomplete laboratory test results. Another study done by Belete Tegbaru on the status of HIV screening laboratories in Ethiopia has also indicated that there are poor laboratory management and lack of follow-up in many laboratories; such problems were also highly seen in the laboratories which participated in this study [11].

## 5. Conclusion and Recommendation

The clinical chemistry test performance shows that coverage in the study area was very low. We also found that the qualities of clinical chemistry tests service given by the health facilities were under the standard that facilities did not fulfill the minimum requirement (less than 1 star service). Therefore, laboratories should have a regular internal quality control program; it will be also advisable for laboratories to report results with the right reference range, so that physicians will refer that, during treatment and follow-up of patients, regional and local health bureau should have similar study in every aspect as it enables them to find the gap in the quality of the test services facilities which are giving. But importantly all stakeholders around the lab service should encourage not only the establishment of local clinical chemistry reference range but also using of it. External quality assessment is also very essential for those health facilities by governmental and nongovernmental agency working towards accreditations. And, these health facilities should also seek accreditation and then should work towards delivering quality of clinical chemistry test results by performing internal quality control daily.

## Figures and Tables

**Table 1:** Distribution of health facilities according to their ownership, location, and reason for not undertaking clinical chemistry tests.

Type of variable		Health facilities without clinical chemistry test service				Total
		Medium clinic	Hospitals	HC	NGO	

Owned by	Governmental	0	2	48	0	50(75%)
	Private	15	0	0	0	15(23%)
	NGOs	0	0	0	1	1(2%)

Residence	Rural part of the Zone	3	2	45	0	50(75%)
	Town	12	0	3	1	16(25%)

Reason for not undertaking clinical chemistry tests	Lack of budget	15	0	45	1	61(92.4%)
	Lack of skilled professional to undertake clinical chemistry tests			3		3(5%)

**Table 2:** Distribution of different factors that are expected to affect the quality of clinical chemistry service among health facilities with clinical chemistry test service.

Type of variable		Health facility with clinical chemistry test service						Total
		Medium clinic	Higher clinic	Hospital	Diagnostic Clinic	Other governmental	NGO	
Score	10-19	5	1	0	1	0	0	7(35%)
	20-29	4	0	0	0	0	1	5(25%)
	30-39	1	0	0	2	1	0	4(20%)
	40-49	0	2	2	0	0	0	4(20%)

Company	Horiba	0	0	1	2	0	0	3(15%)
	Linear	5	3	0	1	0	0	9(45%)
	Human	6	0	0	1	1	0	8(40%)

Analyzer	Humastar 80	1	0	1	0	0	0	2(10%)
	Pentra 400	0	0	1	0	0	0	1(5%)
	Humastar 2000	2	0	0	1	0	0	3(15%)
	Humastar 200	0	0	0	0	0	1	1(5%)
	Linear chemicals	2	2	0	1	0	0	5(25%)
	ARCHITECT c4000	0	0	0	1	0	0	1(5%)
	5010 chemistry analyzer V5+	2	0	0	0	1	0	3(15%)
	Dr.Lange	0	1	0	0	0	0	1(5%)
	Mindray	2	0	0	0	0	0	2(10%)
	ECHO-PLUS	0	0	0	1	0	0	1(5%)

Reagent	Linear	5	2	0	2	0	1	10(50%)
	Human	5	1	0	2	1	0	9(45%)
	ABX- pentra	0	0	1	0	0	0	1(5%)

Personnel	MSc	0	0	0	1	0	0	1(5%)
	BSc	3	3	2	1	1	1	11(55%)
	Diploma	8	0	0	0	0	0	8(40%)

Owned by	Governmental	0	0	2	0	1	0	3(15%)
	Private	10	3	0	3	0	0	16(80%)
	NGO	0	0	0	0	0	1	1(5%)

*∗*Abbreviation: NGO; non-government organization

**Table 3:** Normal range, stated value, target value (manufacturer), mean of participating laboratories, and acceptable values based on TEa ± 20%, ±10%, ±15%, and ±20 for each analytes ALT, glucose, creatinine, and total bilirubin, respectively,from Humatrol P control sample given in IU/L and mg/dl.

Type of test	Normal range	Stated value(author)	Target value (manufacturer)	Mean of participating laboratories	Acceptable range based on stated value	Acceptable range based on mean value
ALT	112.0-180.0	139.7	2.4-142.0	110.1	118.7-167.6	88.1-132.2

Glucose	229.0-317.0	292.5	15.2-273.0	218.4	263.3-321.8	196.6-240.2

Creatinine	3.6-5.6	4.2	4.2-4.8	4.2	3.6-4.9	3.6-4.8

Bilirubin total	3.4-5.8	6.9	72.6-4.3	4.4	5.5-8.2	3.5-5.3

**Table 4:** Normal range, stated value, target value (manufacturer), mean of participating laboratories, and acceptable values based on TEa ± 20%, ±10%, ±15%, and ±20 for each analytes ALT, glucose, creatinine, and total bilirubin, respectively, from Humatrol N control sample given in IU/L and mg/dl.

Type of tests	Normal range	Stated value	Target value	Mean	Acceptable range based on stated value	Acceptable range based on mean value
ALT	24.2-38.7	36.6	21.3-34.0	23.3	29.3-44.0	18.6-28.0

Glucose	92.6-128.0	117.0	134.0-194.0	95.7	105.3-128.7	86.2-105.3

Creatinine	1.1-1.8	1.5	1.3-2.0	1.4	1.3-1.8	1.2-1.7

Bilirubin total	1.1-1.8	2.4	0.6-1.0	1.4	1.9-2.9	1.1-1.7

**Table 5:** Summaries of the results of all test values obtained from two governmental hospitals, two higher clinics, two diagnostic clinics, one other governmental, and three medium clinics laboratories in Jimma zone.

Tests	Sample	Stated value	Mean value	No of values reported	Range	Median	SD	Allowable limit of error (%)	% of unacceptable values	% acceptable values
ALT	P	139.7	110.1	30	50.2	108.0	13.2	20	76.6	23.3
	N	36.6	23.3	29	33.3	25.5	6.9	20	89.7	10.3

Glucose	P	292.5	218.4	30	122.0	217.4	28.15	10	96.7	3.3
	N	117.0	95.7	30	61.0	93.4	12.7	10	83.3	16.7

Creatinine	P	4.2	4.2	24	4.0	4.0	.9	15	41.7	58.3
	N	1.5	1.4	25	0.6	1.4	.16	15	38.0	72.0

Total bilirubin	P	6.9	4.4	24	3.5	4.7	.9	20	95.8	4.2
	N	2.4	1.4	24	2.5	1.1	.64	20	95.8	4.2

Average									77.2	24.0

## Data Availability

The data used to support the findings of this study are available from the corresponding author upon request.

## Abbreviations

AIDS: Acquired immune deficiency syndrome
ART: Antiretroviral therapy
CSA: Central statistics agency
CV: Coefficient of variation
EPHI: Ethiopian public health institute
EQA: External quality assessment
EQC: External quality control
FMoH: Federal Ministry of Health
HIV: Human immune deficiency virus
HSDP: Health service development plan
IQC: Internal quality control
ISO: International Organization for Standardization
NGO: Nongovernmental organization
NMR: Nuclear magnetic resonance
PPM: Part per million
PT: Proficiency testing
QA: Quality assurance
QC: Quality control
SD: Standard deviation
SLIPTA: Stepwise Laboratory (Quality) Improvement Process towards Accreditation
TTP: Total testing process.
